# Opinions on contributing data to research studies: survey results from adults living with juvenile idiopathic arthritis (JIA)

**DOI:** 10.1093/rheumatology/keag331

**Published:** 2026-06-25

**Authors:** Lianne Kearsley-Fleet, Jasmine Leslie, Natasha Shaw, Michelle Johnson, Lucy R Wedderburn, Kimme L Hyrich, Jenny H Humphreys

**Affiliations:** Centre for Epidemiology Versus Arthritis, Centre for Musculoskeletal Research, Division of Musculoskeletal and Dermatological Sciences, The University of Manchester, Manchester Academic Health Sciences Centre, Manchester, UK; Centre for Epidemiology Versus Arthritis, Centre for Musculoskeletal Research, Division of Musculoskeletal and Dermatological Sciences, The University of Manchester, Manchester Academic Health Sciences Centre, Manchester, UK; Centre for Epidemiology Versus Arthritis, Centre for Musculoskeletal Research, Division of Musculoskeletal and Dermatological Sciences, The University of Manchester, Manchester Academic Health Sciences Centre, Manchester, UK; Centre for Epidemiology Versus Arthritis, Centre for Musculoskeletal Research, Division of Musculoskeletal and Dermatological Sciences, The University of Manchester, Manchester Academic Health Sciences Centre, Manchester, UK; Infection, Immunity and Inflammation Research and Teaching Department, UCL Great Ormond Street Institute of Child Health, London, UK; NIHR Biomedical Research Centre at Great Ormond Street Hospital, London, UK; Centre for Adolescent Rheumatology at UCL, UCL Hospital and Great Ormond Street Hospital, London, UK; Centre for Epidemiology Versus Arthritis, Centre for Musculoskeletal Research, Division of Musculoskeletal and Dermatological Sciences, The University of Manchester, Manchester Academic Health Sciences Centre, Manchester, UK; NIHR Manchester Biomedical Research Centre, Manchester University NHS Foundation Trust, Manchester, UK; Kellgren Centre for Rheumatology, Manchester Royal Infirmary, Manchester University Hospitals NHS Trust, Manchester, UK; Centre for Epidemiology Versus Arthritis, Centre for Musculoskeletal Research, Division of Musculoskeletal and Dermatological Sciences, The University of Manchester, Manchester Academic Health Sciences Centre, Manchester, UK; NIHR Manchester Biomedical Research Centre, Manchester University NHS Foundation Trust, Manchester, UK; Kellgren Centre for Rheumatology, Manchester Royal Infirmary, Manchester University Hospitals NHS Trust, Manchester, UK

**Keywords:** patient involvement, data usage, healthcare data, life-course

## Abstract

**Objectives:**

Many childhood-onset chronic conditions continue into adulthood. Life-course research is essential to understand long-term outcomes in these individuals. Parents/guardians consent children into cohort studies and data-linkage with medical records. However, under UK ethics, linkage must stop at age 16 without specific re-consent. This presents challenges, as many young adults have moved, been discharged or changed hospitals. This research aims to understand opinions on contributing data to research studies, particularly continuing to use long-term health outcome data into adulthood.

**Methods:**

A Qualtrics survey was developed alongside patient partners and distributed January 2025 via social media, patient partners and national arthritis charities. Ethical approval was granted by the University of Manchester Research Ethics Committee (2024–21972-38819).

**Results:**

Fifty-seven adults with childhood-onset arthritis completed the survey; 93% were female, 93% were White, 56% were aged ≥45 years, 46% were diagnosed between 11 and 16 years, and 49% had previously participated in a research study. Overall, 88% reported they would have joined an arthritis research study in childhood, and 79% would consent for study data to link with medical records. Most (95%) participants would join a research study as an adult, and 50% were happy for continued access to medical records after age 16 without explicit re-consent and/or assumed it was already happening.

**Conclusion:**

These findings suggest that current consent processes for studies spanning from childhood into adulthood are inadequate. There is a need to re-evaluate consent procedures for life-course research to ensure that paediatric and young people’s research remains a priority, and that young adults with childhood-onset conditions are not unnecessarily excluded from research.

Rheumatology key messages

- Current consent processes for life-course research exclude many individuals from participating, contributing biased research estimates.
- Most individuals with childhood-onset arthritis would contribute to research as a child and into adulthood.
- Key reasons for contributing to research included improving outcomes and building knowledge for future generations.

## Introduction

Many childhood-onset long-term conditions, such as juvenile idiopathic arthritis (JIA), continue into adulthood [1]. However, research into what the impact of a long-term condition is throughout the life-course is limited. Understanding what to expect for the future is a core priority for patients and families. Historically research has been restricted to that of children only [2], or adults with ongoing arthritis only [3], with limited reports of research with outcomes that span childhood into adulthood, regardless of current disease status in adulthood. Continuing research from childhood into adulthood is essential to understand long-term clinical, physical, social and psychological outcomes over the life-course. For children to participate in a research study, parents or guardians must give their consent. To complement active data collection of disease-specific information from hospitals or the patients themselves, in the UK research datasets can be linked with national datasets to supplement study data with additional information from hospital medical records [4].

Continuing follow-up within a study into adulthood is very challenging. There are estimated to be 16 commissioned paediatric rheumatology centres across England [5], with ∼10 times as many adult rheumatology centres nationally [6]. As a result, adult rheumatologists at adult rheumatology centres are likely to see very few patients with childhood-onset arthritis compared with their overall case load. These centres may not be aware of the vital need for this research to continue, they may not want to contribute, or they may be under-resourced with limited research infrastructure to participate, and thus the patient’s outcomes into adulthood are lost. However, even when a rheumatology centre seeing adult JIA patients is happy to continue following them into adulthood, the young person has to re-consent at the age of 16 years old. It disadvantages those already struggling for health equity including those from more deprived areas, or from minority or other under-represented backgrounds [7, 8]. In addition, there is increasing awareness and wariness of scams, i.e. people who falsely contact you to steal your personal data. This means that even if contact information was correct, any communications may be disregarded as ‘fake’. European cohort studies following individuals with childhood-onset arthritis have also found significant attrition of patients into adulthood, particularly as patients that continue to contribute data are limited to only those visiting a clinician [9]. Alternatively adult only cohorts have been developed to support follow-up throughout adulthood [10], although starting new cohorts is resource demanding and may still miss individuals lost between the transition from paediatric to adult care, a vulnerable age group that tend to stop visiting healthcare professionals [5]. Therefore, due to the fact that, in many countries, research into these individuals cannot continue without explicit consent from the age of 16 years, further data capture of outcomes is very challenging.

In the UK, through linkage with national hospital medical record datasets, even if a patient has been discharged from clinic in remission, or moved house or hospital, it would be possible to track future visits to a rheumatologist, or other hospital speciality (e.g. joint replacements). This information is invaluable, particularly as some of the main questions asked from patients and families are whether their arthritis will ‘come back’ after going into remission as a child, or whether they will ‘grow out of it’ as they grow up, and in which patients is remission or relapse more likely to occur, for which we do not have the answer. In addition, access to these medical databases allows us to understand healthcare utilization or burden, development of comorbidities into adulthood, impact of arthritis on development of cancer or mortality. However, as with the core research data, initial data linkage in a paediatric study is consented to by the parent or guardian, and under current UK ethics, linkage of data must stop at the age of 16 years old unless the person re-consents themself. This means that without continued follow-up of all participants into adulthood, research into longer term outcomes in these individuals becomes impossible.

It is possible to get special permissions to link data without specific consent through the Health Research Authority (HRA) Confidentiality Advisory Group (CAG) [11]. However, this does not guarantee permission to the data, and is considered a temporary fix whilst long-term methods to gain re-consent must be implemented for all ongoing studies. This is not an easy option for ongoing life-course research studies, particularly when many children may have left the study prior to adulthood or can be difficult to locate within the health care system after transition to adult rheumatology. Instead, individuals must re-consent themselves at the age of 16 years old for this data linkage to continue. This risks significant loss of data and high risk of selection bias among those who do consent.

The aim of this research is to understand people’s opinion on contributing data to research studies, particularly on researchers continuing to use long-term health outcome data into adulthood with historical consent from parents or guardians alone (i.e. without the participant re-consenting at the age of 16 years old).

## Methods

### Survey development

A survey was developed alongside patient partners to capture how people with childhood-onset arthritis felt about participating in research studies, contributing data from their medical records, and whether they would continue to participate in research studies as adults (see Supplementary Data S1). Adults previously diagnosed with childhood-onset arthritis and living in the United Kingdom (UK) were eligible to complete the survey. Information collected included demographic data (age, gender, ethnic group, arthritis disease duration, UK nation, settlement), views on joining a research study as either a child or an adult, views on data linkage to external national datasets, and whether they would be happy for the research team to continue to access these data in adulthood without asking for their explicit re-consent.

The survey, consent forms and patient information sheets were written in English. The survey was uploaded to Qualtrics with links to the patient information sheet. The link to the Qualtrics survey was distributed week beginning 6 January 2025 via (i) social media (professional account: L.K-F.), (ii) patient partners, and (iii) national arthritis charity email lists.

This study was approved by the University of Manchester Research Ethics Committee (UREC): 2024–21972-38819.

### Data analysis

Only participants who confirmed that they had arthritis diagnosed in childhood and were happy to participate were included for analysis. Results are presented as proportions, including free text quotes where applicable. Data were analysed using Stata 14 (StatCorp. LLC, College Station, TX, USA).

### Patient and public involvement statement

This work has been supported by three patient partners, all co-authors on the manuscript.

## Results

As of 10 March 2025, a total of 68 responses were received to the online survey. Of these, 61 confirmed that they had arthritis diagnosed in childhood and were happy to proceed. A further four participants answered demographic information but did not answer the questions about their views on research studies and data linkage; these were excluded from the analysis.

### Participant demographics

Of the 57 participants included (Table 1), over half (56%) were aged 45 years old or over, with 21% aged between 35 and 44 years, 16% between 25 and 34 years, and 7% between 16 and 24 years old. Most had been diagnosed either between 11 and 16 years old (46%) or under the age of 5 years old (37%), with 18% diagnosed between the age of 5 and 10 years old. The majority were female (93%), of White ethnicity (93%) and living in England (91%), with 46% living in a town, 30% a village or hamlet, and 25% a city. Overall, 27 participants had participated in a research study (45% of those individuals aged 35 and over, and 64% of those individuals aged <35 years), either as an adult (*n* = 16), a child (*n* = 6) or both (*n* = 5).

**Table 1:** Survey participant responses of 57 adults with childhood-onset arthritis.

	Included participants
**Total number**	57
**Patient demographics**	
**How old are you?**	
Aged 16 − 24 years old	4 (7%)
Aged 25 − 34 years old	9 (16%)
Aged 35 − 44 years old	12 (21%)
Aged 45 years old or over	32 (56%)
**What is your gender?**	
Female *vs* male/non-binary/third gender/prefer not to say	53 (93%)
**What is your ethnic group?**	
White *vs* mixed/Asian/black/other	53 (93%)
**What age were you first diagnosed with arthritis?**	
Under 5 years old	21 (37%)
Between 5 and 10 years old	10 (18%)
Between 11 and 16 years old	26 (46%)
**Where do you live?**	
England *vs* Wales/Scotland/Northern Ireland	52 (91%)
**What type of settlement do you current live in?**	
City	14 (25%)
Town	26 (46%)
Village/hamlet/middle of nowhere	17 (30%)

**Joining research studies**	
**As a child, would you have wanted to join an arthritis research study (with parental/guardian consent)?**	*n* = 57
Yes	50 (88%)
No	4 (7%)
More information needed	3 (5%)
**As a child, would you have also *consented* for the research team to link your research study data with your national medical records (alongside your parental/guardian consent)?**	*n* = 57
Yes	45 (79%)
**If also answered yes above (*n* = 50)**	43 (86%)
No	6 (11%)
More information needed	6 (11%)
**Would you consider continuing to contribute information to this research study now as an adult?**	*n* = 56
Yes	53 (95%)
No	1 (2%)
More information needed	2 (4%)
**Would you be happy for the research team to *continue to access your national medical records* now that you are an adult, *without asking you for your explicit consent*? [multiple options]**	*n* = 56
No—I would want to make the decision to consent myself	25 (45%)
Yes—I would be happy for the research team to continue using my data based on my parental/guardian consent, without the need for me to consent as an adult personally	20 (36%)
Assumed—I would have assumed that long-term data was already being collected on me based on the original parent/guardian consent—I don’t understand why I would need to re-consent myself now I am over 16 years old	14 (25%)
More information	5 (9%)
**Both yes and assumed (excluding any ‘no’ answers)**	28 (50%)
**Have you ever been involved in a research study before?**	
**As a child**	*n* = 54
Yes	11 (20%)
No	34 (63%)
Maybe	9 (17%)
**As an adult**	*n* = 55
Yes	21 (38%)
No	28 (51%)
Maybe	6 (11%)
**As either child or adult**	27/55 (49%)
**Child only**	6 (22%)
**Adult only**	16 (59%)
**Both**	5 (19%)

### Joining research studies as a child

A total of 50 (88%) participants reported that they would have wanted to join an arthritis research study as a child (with their parent’s or guardian’s consent). This altered with age of the participant, with 91% of those aged 35 and over responding that they would want to join, compared with 77% of under 35-year-olds. The key reasons stated for this included to better understand their condition, to contribute to research, to improve future treatments, and personal fulfilment. Three of the participants reporting no (or needing more information) reported that they would have been too young to personally understand what a research study was, feared having more visits or tests, or that it was over 40 years ago with less awareness at that time for the importance of research.

In addition, most of the participants (79%) said that if they were part of a research study as a child they would have also consented for the researchers to link the study data with their medical records—89% of those aged 35 and over, and 46% of those under 35 years old. Two of the participants responding no (or needing more information) highlighted that they wouldn’t have understood the meaning of this, or that when they were children this opportunity was not available, although neither indicated their view had it been a possibility or had they understood what the data linkage meant. A further two participants were concerned about lack of control over their data or their data being sold to third parties.

### Continuing to contribute data to that research study as an adult

When thinking about childhood-onset arthritis research studies, of the 56 participants that responded, 53 participants (95%) reported that they would consider contributing information to this study as an adult—98% of those aged 35 and over, and 83% of those under 35 years old. Reasons mirrored those given for joining a study as a child, including to help themselves and others with the condition, improve outcomes and find effective treatments, and to build knowledge for future generations. One of the participants responding no (or needing more information) reported that ‘as a child you [get] the research for a child with JIA so no longer needed’. This reinforces that there is a lack of information available to people that JIA can continue into adulthood and that research in this area is needed.

Finally, when looking at whether participants would be happy for the research team to continue to access national medical records after the age of 16 years old without asking for explicit consent from participants (relying solely on original parental or guardian consent), 50% responded that they would be happy with this. Proportions were similar between age groups, with 48% of those aged 35 and over, and 58% of those <35 years old responding they would be happy for this linkage to continue. Comments from participants reiterated previous responses to reasons for contributing their data to research studies. In addition, 25% of the participants who responded assumed that the linkage was already continuing into adulthood. Participants highlighted they should ‘already be aware of their participation in the study and [would be] happy for it to continue into adulthood’, with some suggesting participants should not be contacted for re-consent at age 16 years, but rather with ‘an option to withdraw’. One participant commented ‘I would have thought [that] research only up to 16 would limit things for those with long term conditions’.

However, 25 participants (45%) reported that they would not want linkage to continue without their explicit consent; proportions were similar between age groups, and by whether they had previously participated in research studies before. Reasons included wanting autonomy, importance of communication updates with research team, and awareness of what was happening with their data.

## Discussion

This scoping survey of adults with childhood-onset arthritis aimed to understand the opinion of people with lived experiences on contributing data to research studies. In particular, whether individuals would want to continue contributing long-term health outcome data into a research study from childhood into adulthood using historical consent from parents or guardians (i.e. without the participant having to re-consent at the age of 16 years old). Overall, 95% of the respondents were happy to join a research study and continue contributing information into the study into adulthood. In addition, when asked whether they would want this data collection to continue despite the researchers being unable to contact them for their explicit re-consent at the age of 16 years old, 50% were happy with this, with many assuming that if they were part of the research study, data collection was already continuing into adulthood regardless. Key reasons highlighted among those who wanted to contribute their data were focused around being able to help themselves and others with the condition, improve outcomes, find effective treatments, and build knowledge for future generations. One participant highlighting that people who are part of a childhood chronic condition cohort study should ‘already be aware of their participation in the study and [would be] happy for it to continue into adulthood’.

A large societal barrier for only including participants with contact information available, who have not been discharged or transferred to adult services already, is that the information collected from the remaining population is a biased cohort. These are the individuals with the most severe disease, visiting hospitals that are often tertiary centres and better resourced to collect research data. In addition, contact information may change from the age of 16 years old and onwards as they move hospital or geographically for careers, meaning they may not always be up-to-date, or even if they are, contact may be mistaken for a scam. There were concerns from some participants about the lack of control over their data, or their data being sold to third parties. This is always specifically addressed in study information leaflets explaining what happens to the data and who has access. However, this is provided at the start of the research study, and it is understandable that as participants continue to contribute over time that this information is forgotten about. This is why, as suggested by one of the survey participants, communication updates with the research team either through their rheumatology clinics or via any personal contact information for patients and families within studies are useful to report study updates. This method to keep study participants informed and engaged, and developing an awareness of the research study and trust with the research team, is something already completed by most studies, although rather than focusing solely on research results, research teams could also reiterate data security and governance.

Based on discussions with young adults with childhood-onset arthritis and charity partners, it is clear that there is a significant information gap on what happens to people with childhood-onset arthritis during adulthood, resulting in a lack of support for these individuals and their families. Patient partners (co-authors on this manuscript) have highlighted that ‘disease activity (i.e. how active someone’s arthritis is) and its effect on the day-to-day lives of those with JIA doesn’t stop at the age of 16 years old’. Therefore, they would assume that if they participated in a research study as a child, that data collection would continue into adulthood. They highlighted that it is difficult enough as a 16-year-old, having to change doctor and potentially hospital, where information notoriously ‘gets forgotten, treatment and care levels change’, and inclusion into research studies are ignored or deprioritized. ‘Therefore, when part of an important research study which aims to investigate what happens to people like me with JIA, I would hope that there would be an automatic continuation of data [collection] to at least maintain consistency and provide valuable information that we all want’. In addition, many of the national arthritis charities highlight that after discussions with their patients and families, the majority assume that research into adulthood continues, particularly as the consent forms do not state that it will have to stop at the age of 16 years old. They are surprised and disappointed to hear that their data are not being used in a way they believe they want and consented for. There is an awareness that in a perfect world everyone would be able to re-consent, but there is a realistic expectation that this is not always possible, and that alternative options should be considered. Through excluding individuals where only prior parental/guardian consent was achieved (not additional patient re-consent), is not only ‘in direct contrast to their wishes’, but means ‘that any estimates calculated will be biased against “at risk” patient groups who [are] excluded from the analyses’. The charities felt strongly that the patients and families ‘want to see the data and insights they provide used to improve outcomes and understanding—for both themselves as well as the next generation of children diagnosed’.

Awareness and autonomy of data is of key importance. One survey responder suggested that research study participants should not be contacted for re-consent at the age of 16 years old, but rather with ‘an option to withdraw’. This would revolutionize life-course epidemiological studies into conditions starting in childhood as it would remove any requirement for participants to re-consent. Research results would no longer be biased through the exclusions of those in remission, attending under-resourced hospitals with limited research infrastructure, from more deprived areas, or from minority or other under-represented backgrounds. Instead, all individuals would be included, and research outputs would be more relevant to the whole population.

The UK already has an ‘opt out’ scheme for patient consent with respect to their electronic health records, which means that research can be representative throughout the life-course [12]. Currently, researchers can apply to use these data for their research without direct consent from the patients, meaning that all patient data is being accessed and used. However, for disease-specific cohort studies (where separate ethics for research is required), despite the family providing initial consent to participate, this continuation of data into adulthood is prohibited. Instead, no data access is allowed beyond the age of 16 years old without the person’s explicit consent despite consent already having been provided by the family in childhood and the participant already contributing data. Mismatch between cohort studies *vs* national datasets unfairly penalizes paediatric and young people’s research, which, despite having the potential to provide rich valuable data regarding conditions across the life-course, is currently absent for individuals once they become adults. One participant commented ‘I would have thought [that] research only up to 16 would limit things for those with long term conditions’. We propose that the process for consenting children into long-term studies should be re-evaluated to ensure that young adults are not unnecessarily excluded from research. Through introducing an opt-out clause to all life-course research studies, research participants retain the control and awareness of their own data, whilst simultaneously ensuring that research outputs are as unbiased and relevant as possible for those who need it. This would then align these research studies with those that use routinely collected data alone, where the opt-out scheme already is in place. Any data outputs would be required to adhere to the highest standards of data protection, as outlined in the Five Safes Framework (supported by the Health Data Research-UK and National Institute for Health Research Design Service) to ensure no re-identification of individuals [13].

This survey represents the views of people with childhood-onset arthritis in the UK who wanted to take part in the survey about their views on research using their data. Participants were predominantly females of white ethnicity living in England, who received this survey via social media, the patient partners or charity email lists. It may not be generalizable to the whole of the UK, nor other countries. The proportion of older (≥45 years old) to young participants was similar indicating the responses are applicable to most ages, although the proportion of very young participants (under 25 years old) was small, meaning the opinions of the younger generation cannot be concluded. However, the views presented by the survey participants are invaluable and mark an important step to ensure that research is both fair and inclusive. It would be particularly useful moving forward to explore qualitative research to understand further what the motivators behind these patient views are, and what the barriers are for consent and data usage across the life-course. Considering the majority of the respondents would have wanted to contribute to a research study as a child and also into adulthood, it was somewhat surprising that 45% responded that they would not want the research team to continue to collect their health data after the age of 16 years old without their explicit consent. Some reasons highlighted for this were around wanting autonomy, importance of communication updates with the research team, and awareness of what was happening with their data. Many participants also commented that their GP or rheumatologist could contact them for their re-consent, despite the survey highlighting that some studies do not collect personal contact information and therefore contacting for re-consent is not possible. The wording of the survey asked about ‘continued access to national medical records without asking for your explicit consent’. It is possible, that this was interpreted as having no consent from patients and families whatsoever. Instead, it is important to highlight that consent (or assent) was already received for participation into the study initially, and the question in this survey was aimed at re-consenting individuals to *continue* contributing within the study as an adult. Additional qualitative research to understand why there are these discrepancies and what the barriers really are for consent and data usage are needed.

In conclusion, this survey identified that almost all those with childhood-onset arthritis would want to continue to participate in a research study from childhood into adulthood, i.e. throughout the life-course. Many assumed that this continued contribution would automatically happen and seemed concerned at this resulting research gap. This highlights the need to re-evaluate consent procedures for research studies that span both childhood and adulthood, across the life-course, considering an option to opt-out rather than re-consent at the age of 16 years old. Without this, life-course research ceases at the age of 16 years old unless the participant can be re-consented, biasing research results to exclude those who have left or moved hospitals, attending under-resourced hospitals, or those already struggling for health equity including those from more deprived areas, or from minority or other under-represented backgrounds. It is important to ensure that paediatric and young people’s research remains a priority, that young adults with childhood-onset conditions are not unnecessarily excluded from research, and that research outputs are relevant to the whole population.

## Supplementary Material

keag331_Supplementary_Data

## Data Availability

The data used in this survey is not available for use outside of the research team.
