# Supplementary material for: Opinions on contributing data to research studies: survey results from adults living with juvenile idiopathic arthritis (JIA)
Source: Rheumatology (Oxford). 2026 Jun 25;65(7):keag331. doi: 10.1093/rheumatology/keag331 (PMC13354604; doi:10.1093/rheumatology/keag331)
Supplement: keag331_Supplementary_Data [file keag331_supplementary_data.docx]

Supplementary Materials

**Supplementary Data S1: Survey**

Patient Views on Continued Data Linkage

**This survey is for adults living in the United Kingdom who received a diagnosis of arthritis in childhood (under 16 years of age).**

The patient information sheet (version 2; 18-Dec-2024) should be read before continuing.

**If you are happy to participate, please tick all consent form statements below (all must be ticked):**

- I confirm that I have read the attached information sheet (Version 2, Date 18-Dec-2024) for the above study and have had the opportunity to consider the information and ask questions and had these answered satisfactorily.
- I understand that my participation in the study is voluntary and that I am free to withdraw at any time without giving a reason and without detriment to myself. I understand that it will not be possible to remove my data from the project once it has been anonymised and forms part of the data set. I agree to take part on this basis.
- I agree that any data collected may be included in anonymous form in publications/conference presentations.
- I agree that any research publications can include direct quotes of my responses in anonymous format.
- I understand that data collected during the study may be looked at by individuals from The University of Manchester or regulatory authorities, where it is relevant to my taking part in this research. I give permission for these individuals to have access to my data.
- I agree to take part in this study.

**Data Protection**

The personal information we collect and use to conduct this research will be processed in accordance with UK data protection law as explained in the Participant Information Sheet and the Privacy Notice for Research Participants.

Currently in Research:

Currently when children join research studies, it is their parent or guardian who gives consent. For researchers to continue to collect data beyond the age of 16 years old, they need to obtain consent from the young adult (participant) directly. In addition, researchers are keen to learn about lots of other outcomes already collected which they may need to get directly from NHS records rather than asking the person or their doctor directly. This is because the NHS keeps very good records of every hospital admission, cancer or death that occurs so they can also learn more about the health of the nation. Therefore, an option in research is for researchers to link, through your NHS number, your participation in the study with these other national databases. This will give them additional information on your health outcomes and allows for a better understanding of how treatments work and any long-term effects. For researchers to be able to link to your NHS records, some **personal confidential information** (name, date of birth, gender, postcode, NHS Number) already collected by the research study, **is shared** between the research study and NHS England (who hold your data). After this step, any health data then returned to the researchers are stored securely.

The Aim of this Survey:

The aim of this survey is to understand your opinion, as a child and as an adult, on research studies. We are particularly keen to understand your opinion on researchers continuing to use your long-term health outcome data into adulthood with historical consent from your parent / guardian alone (i.e. without you re-consenting at the age of 16 years old).

*This survey is anonymous and will not be traced back to you. Please share your honest opinions. There is no right or wrong answer.*

**Please confirm the following:**

- I had arthritis in childhood, i.e. JIA (regardless of whether you still have symptoms or are now in remission)
- I am over 16 years old
- I live in the United Kingdom
- I am happy to proceed with the survey

**Firstly, we would like to know more about you** - so that we can ensure we are collecting views from many different people across the country.

**How old are you?**

- Aged 16-24 years old
- Aged 25-34 years old
- Aged 35-44 years old
- Aged 45 years old or over

**What is your gender?**

- Male
- Female
- Non-binary / third gender
- Prefer not to say

**What is your ethnic group?**

- White (including English / Welsh / Scottish / Northern Irish / British, Irish, Gypsy or Irish Traveler, or any other White background)
- Mixed/Multiple ethnic groups (including White and Black Caribbean, White and Black African, White and Asian, or any other Mixed/Multiple ethnic background)
- Asian/Asian British (including Indian, Pakistani, Bangladeshi, Chinese, or any other Asian background)
- Black/ African/Caribbean/Black British (including African, Caribbean, or any other Black/African/Caribbean background)
- Other ethnic group (including Arab, or any other ethnic group)

**At what age were you first diagnosed with arthritis?**

- Under 5 years old
- Between 6 and 10 years old
- Between 11 and 16 years old

**Where do you live?**

- England
- Wales
- Scotland
- Northern Ireland

**What type of settlement do you currently live in?**

- In the middle on nowhere / hamlet
- In a village
- In a town
- In a city

Imagine you are a child again, and following your diagnosis with arthritis, your parent or guardian signed you up for a long-term (observational) research study and signed the consent form on your behalf. This study would collect information from your doctor (rheumatologist) about your arthritis, your well-being, any medication you are on, and any side effects. It is information that the doctor collects routinely when you visit them in clinic – no extra visits, tests or treatments are needed. The aim of the study is to establish what the impact of your arthritis symptoms and arthritis treatments are on you and others like you.

**As a child, would you want to join an arthritis research study (with parental / guardian consent)?**

- No (please give a reason) _________________________________________________
- I would like more information
- Yes (please give a reason) ________________________________________________

In addition, researchers are keen to learn about lots of other outcomes already collected about you which they may need to get directly from your NHS records rather than asking your doctor. This is because the NHS keeps very good records of every hospital admission, cancer or death that occurs so they can also learn more about the health of the nation.

Therefore, an option in research is for researchers to link your participation in the study, through your NHS number, with these other national databases. This will give them additional information on your health outcomes and allows for a better understanding of how treatments work and any long-term effects.

For researchers to be able to link to your NHS records, some **personal confidential information** (name, date of birth, gender, postcode, NHS Number) already collected by the research study, **is shared** between the research study and NHS England (who hold your NHS records). After this step, any health data then returned to the researchers are stored securely.

**As a child, would you have also consented for the research team to link your research study data with your NHS records (alongside your parental / guardian consent)?**

- No (please give a reason) _________________________________________________
- I would like more information
- Yes (please give a reason) ________________________________________________

**We are now interested to know what you think about researchers continuing to use your data after you turn 16 years of age.**This is because you would now be classed as an adult, and the consent form signed by your parent or guardian originally would no longer be valid. All adults need to consent to participate in research studies for themselves as it is considered unethical to continue based solely on parental / guardian consent alone.

Imagine you were in a research study as a child. You have recently turned 16 years old, and the researchers are contacting you to see if you still wanted to be involved in the study. The aim of this research study is to investigate how having arthritis since childhood may impact you and others like you into adulthood.

**Would you consider contributing information to this research study as an adult?**

- No (please give a reason) _________________________________________________
- I would like more information
- Yes (please give a reason) ________________________________________________

Continue to imagine that you were in a research study as a child, and you are now over the age of 16 years old. However, it is possible that you have been in remission for a while and have been discharged from regular hospital visits, or are now with a different adult rheumatology hospital, and are therefore no longer in contact with your original paediatric rheumatologist. This means that the research team have **no contact details for you and no way to contact you** to see if you would like to continue to be involved in this research study.

The research study could continue to collect data from your adult NHS records, i.e. data that is already collected on you regarding hospital visits, cancer, death. This would mean that the researcher could investigate how having childhood arthritis is impacting people as adults without you having to re-consent to the study yourself.

For researchers to be able to link to your NHS records, some **personal confidential information** (name, date of birth, gender, postcode, NHS Number) already collected by the research study, **is shared** between the research study and NHS England (who hold your NHS records). After this step, any health data then returned to the researchers are stored securely.

**Considering your parent / guardian initially consented on your behalf to take part in this research study during childhood…**

**Would you be happy for the research team to continue to access your NHS records now that you are an adult, without asking you for your explicit consent?**

*(multiple options available)*

- No - I would want to make the decision to consent myself (please give a reason) __________________________________________________
- I would like more information
- Yes - I would be happy for the research team to continue using my data based on my parental / guardian consent, without the need for me to consent as an adult personally (please give a reason) __________________________________________________
- I would have assumed that long-term data was already being collected on me based on the original parental / guardian consent - I don't understand why I would need to re-consent myself now I am over 16 years old (please give a reason) __________________________________________________

We just have one final question:

**Have you ever been involved in a research study before?**

|  | No | Maybe | Yes |
| --- | --- | --- | --- |
| As a child |  |  |  |
| As an adult |  |  |  |

This aim of this survey is to gather views on using data from people after the age of 16 years old, without their explicit consent, i.e. they have not consented themselves since they turned 16 years old (instead continue to use the original consent from their parent / guardian).

If you have any additional thoughts or comments you would like to share with the research team, please insert them below. *Do not include any personal identifiable information.*

________________________________________________________________

________________________________________________________________

________________________________________________________________

________________________________________________________________

________________________________________________________________

**Thank you for your time in completing this survey.** If you have any questions about this survey, or are interested to learn more about the project, please feel free to contact us:

**Lianne Kearsley-Fleet**

Versus Arthritis Career Development Fellow, The University of Manchester

[Lianne.Kearsley-Fleet@manchester.ac.uk](mailto:Lianne.Kearsley-Fleet@manchester.ac.uk)

Thank you for participating in this online survey. We hope that you have found it interesting and have not been upset by any of the topics discussed.

If you have any queries about the study or wish to speak with a member of the research team, please contact: **Lianne Kearsley-Fleet**, 01612751646, [lianne.kearsley-fleet@manchester.ac.uk](mailto:lianne.kearsley-fleet@manchester.ac.uk)

If you have found any part of this experience to be distressing, please consider speaking with someone that you trust such as a family member or friend. You may also consider speaking with one of the organisations below for additional support:

- 42nd Street (a reputable charity offering a social work and counselling service for under 25s): <http://42ndstreet.org.uk>
- 24 hours access <https://www.samaritans.org/how-we-can-help/contact-samaritan>
- Free counselling on the NHS <https://www.nhs.uk/service-search/find-a-psychological-therapies-service>
- Self Help Services (works actively with people in the Greater Manchester area on a number of difficulties including anger, anxiety and depression): <https://www.selfhelpservices.org.uk>

**Please click next to submit your responses.**
